# Variety Screening and Characterization Analysis of Storage Stability of Eating Quality of Rice

**DOI:** 10.3390/foods13244140

**Published:** 2024-12-20

**Authors:** Jinyu Tian, Guangmei Ji, Jiafeng Zhang, Danqiu Luo, Fang Zhang, Lijiang Li, Mingjin Jiang, Dawei Zhu, Min Li

**Affiliations:** 1 Rice Research Institute of Guizhou Province, Guizhou Academy of Agricultural Sciences, Guiyang 550025, China; jinyu230811@163.com (J.T.); jiaguangmei2008@126.com (G.J.); 13688521449@136.com (J.Z.); ldq18198101384@163.com (D.L.); 15329577052@163.com (F.Z.); lljiang8421@163.com (L.L.); mj_jiang2008@163.com (M.J.); 2 Rice Product Quality Supervision and Inspection Center, Ministry of Agriculture and Rural Affairs, China National Rice Research Institute, Hangzhou 310006, China

**Keywords:** rice, eating quality, storage stability, variety screening, characterization analysis

## Abstract

To screen rice varieties with high storage stability for eating quality and elucidate their traits, 34 widely grown rice varieties were selected to examine the changes in the eating quality of their grains after natural storage for one year. A hierarchical analysis, normalization method, and cluster analysis were used to identify the rice varieties that maintained their eating quality during storage. Meanwhile, the yield and its components, panicle traits, grain size, grain major component content, physiological indicators (such as antioxidant enzyme activity), and key growth stages were analyzed at rice maturity. The results showed that after storage, the values of the appearance, texture, and taste of the cooked rice decreased by 18.7%, 19.1%, and 14.2%, respectively. The storage stability of the eating quality of rice was evaluated using a hierarchical analysis based on the storage stability scores of the appearance, texture, and taste of the cooked rice. A judgment matrix was established, where the corresponding weights of the appearance, texture, and taste of the cooked rice were identified to be 0.105, 0.259, and 0.637, respectively. Based on a cluster analysis and the normalization method, these varieties were classified into three categories regarding the storage stability of their eating quality: high storage stability, intermediate storage stability, and low storage stability, accounting for 14.7%, 52.9%, and 32.4%, respectively. Finally, five rice varieties with high storage stability were screened. These varieties exhibited storage stability scores of the appearance, texture, and taste of cooked rice that were 215%, 219%, and 340% higher than those of the low storage stability type, respectively. The correlation analysis revealed that the storage stability of the eating quality of the rice was significantly and negatively correlated with amylose starch content, peroxidase activity, and malondialdehyde content. The amylose starch content, peroxidase activity, and malondialdehyde content of the high storage stability type were 12.4%, 35.9%, and 6.42% lower than those of the low-storage-stability variety, respectively. Therefore, the major features of rice varieties with high storage stability included low amylose starch content, diminished peroxidase activity, and low malondialdehyde content. This study provides valuable theoretical insights into the safe storage of rice grains and the selection and breeding of rice varieties with high storage stability.

## 1. Introduction

Rice is one of the most important staple food crops globally, consumed by more than half of the world’s population [1]. After harvesting, rice grains are typically stored for a period to meet year-round consumption needs [2]. In China, the average storage duration for rice grains is approximately 16 months [3]. However, rice grains are living organisms that generally undergo biochemical reactions, such as respiration. These processes, along with environmental factors, can cause a deterioration in the eating quality of rice grains [4,5]. Therefore, maintaining and improving the stability of eating quality during storage is crucial for ensuring a sustained supply of high-quality rice.

The storage stability of rice grains is a multifaceted trait influenced by numerous factors, such as genetic characteristics [6,7], storage methods [8], and environmental conditions [9]. Rice varieties of different genotypes exhibit varying levels of storage stability. Compared with japonica rice, indica rice maintains a more stable starch fine structure and physicochemical properties after storage. Physiological and biochemical indices, such as lipoxygenase (LOX) activity, peroxidase (POD) activity, superoxide dismutase (SOD) activity, malondialdehyde (MDA) content, and α-amylase (α-AL) activity, were identified as key factors influencing the storage stability of rice grains [10,11]. Notably, the malondialdehyde content tends to increase, and antioxidant enzyme activities decrease significantly after rice storage [12]. Some studies suggest that lipoxygenase-deficient rice varieties exhibit improved storage stability, contributing to better quality retention during storage [13]. Storage under nitrogen gas conditions was found to reduce changes in the germination rates, α-amylase activity, free sulfhydryl content, fatty acid value, electrical conductivity, microstructure, and pasting properties of rice grains compared with conventional storage [14,15]. Low-temperature storage is generally more effective in maintaining lower fatty acid values and a stable eating quality of rice grains than high-temperature storage conditions [16]. High temperature and high humidity during storage negatively impact some groups in the primary starch structure, resulting in changes in the internal arrangement of starch molecules and ultimately affecting the eating quality of rice grains [17].

Many studies explored the changes in the eating quality of rice during storage, focusing on different varietal types or storage conditions. However, there is limited research specifically targeting rice varieties with eating quality storage stability. Additionally, there is a lack of indicators that can accurately reflect this trait. In this study, 34 rice varieties widely cultivated in rice production areas were selected as experimental materials to investigate the changes in eating quality after natural storage for one year, analyzing the characteristics of rice at maturity. The objective is to identify rice varieties that are resistant to deterioration in eating quality during storage and elucidate their storage stability characteristics. This study provides valuable insights for selecting and producing rice varieties that are resilient to quality loss during storage.

## 2. Materials and Methods

### 2.1. Experimental Material

Thirty-four widely grown rice varieties were chosen as the experimental materials: GY725, FY498, CY6203, CY3727, YXY2115, YX3728, R18Y2348, QXY19X, LY4923, DY4923, SY127, N5Y39, FY609, YX203, HY528, TXY557, JY127, TYHZ, ZZY8H, YLY585, XLY619, XLYGFZ, YXYLS, ZY169, YLY1H, XZY2017, GY325, TY808, TY390, QY35, YXYHS, FYXZ, JLYHZ, and JLY534 (Supplemental Table S1).

### 2.2. Experimental Site and Weather Conditions

**Figure 1:**
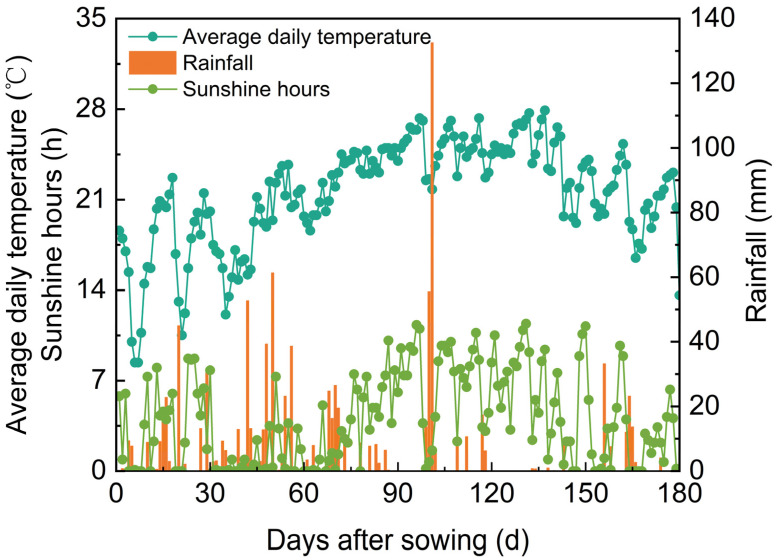
Average daily temperature, sunshine hours, and rainfall during the rice growth season in 2022.

The experiment was conducted in 2022 at the experimental farm of Guizhou Rice Research Institute (altitude: 1150 m, latitude: 26°41′ N, and longitude: 106°66′ E). The previous crop was a winter fallow field, and the soil was classified as yellow loam. The physical and chemical properties of the soil in the tillage layer were 13.9 g/kg of organic matter, 1.20 g/kg of total nitrogen, 86.7 mg/kg of alkaline dissolved nitrogen, 32.8 mg/kg of quick-acting phosphorus, 87.7 mg/kg of quick-acting potassium, and a pH of 6.22. The average daily temperature, sunshine hours, and rainfall of the rice planting season are shown in Figure 1.

### 2.3. Experimental Design

The experimental rice varieties were sown on April 11 and transplanted on May 25 with a planting spacing of 20 cm × 30 cm. During the growing period, 150 kg/ha of N was applied, divided as follows: basal fertilizer (40%) applied one day before transplanting, tillering fertilizer (30%) applied ten days after transplanting, and panicle fertilizer (30%) applied at the inverted 4-leaf stage. Additionally, 75 kg/ha of P was applied as a basal fertilizer, and 150 kg/ha of K was applied as a combination of basal fertilizer (50%) and panicle fertilizer (50%). Pest and weed management were implemented according to the local conventional practices for high-yielding cultivation. There were three replications in the field for each variety.

Upon harvest, the rice grains of the 34 selected varieties underwent impurity separation and were then naturally air-dried. Subsequently, each sample was placed into an individual mesh bag and hung for storage within the warehouse of the experimental base. This warehouse had ventilation facilities that maintained a natural environment without temperature control, nitrogen gas conditioning, or vacuum packaging capabilities. Throughout the one-year storage period, the temperature and other environmental conditions within the warehouse fluctuated naturally and realistically simulated the non-intervention storage conditions that farmers or small-scale grain storage operators might face. Regular inspections were carried out during storage to monitor any potential quality issues, such as mold growth, pest infestation, or abnormal odors to ensure the integrity and reliability of the experimental data. Detailed records of each inspection were maintained for the subsequent data analysis and results verification.

### 2.4. Sampling and Measurement

#### 2.4.1. Eating Quality

The eating quality of the rice grains before storage (fresh rice) and after one year of storage (stored rice) was evaluated using a rice taste analyzer (Satake Column Co., Higashi-Hiroshima, Japan). It includes three key aspects of cooked rice: appearance, texture, and taste. The determination method followed the National Standard of the People’s Republic of China (GB/T17891-2017, High-Quality Rice) [18].

#### 2.4.2. Grain Yield and Its Composition

The number of panicles was counted by surveying 100 holes within each plot at maturity. The grain number per panicle and panicle length were measured from 10 holes in each plot according to the average tiller. The grain weight was recorded at maturity. The yield was obtained by weighing 100 holes harvested per plot at maturity and converted to a standard moisture content of 13.5%. Grain density = grain number per panicle/panicle length [19].

#### 2.4.3. Key Growth Stages of Rice

The dates of sowing, flowering, and maturity for each rice variety were recorded in detail.

#### 2.4.4. Grain Size

The grain length, grain width, and length–width ratio were determined using a rice appearance quality tester (Wanshen Testing Technology Co., Ltd., Hangzhou, China).

#### 2.4.5. Major Component Content in Grains

The protein content, amylose content, amylopectin content, and total starch content of the grains were quantified based on the national standard of the People’s Republic of China (GB/T17891-2017, High-Quality Rice Grain).

#### 2.4.6. Physiological Indicator

At maturity, 200–300 grains were randomly selected and placed in liquid nitrogen for one minute. Then, the grains were stored in an ultra-low-temperature freezer for further analysis of the physiological indicators. The POD activity was determined using the guaiacol method [20], the SOD activity was measured using the nitrogen blue tetrazolium photochemical reduction method [21], the MDA content was quantified by the thiobarbituric acid method [22], the LOX activity was determined using the fluorescence method [23], and the α-AL activity was assessed by the 3,5-dinitrosalicylic acid method [24].

### 2.5. Formulas and Statistical Analysis

#### 2.5.1. Variable Amplitude of the Eating Quality of Rice

The variable amplitude of the eating quality of cooked rice = [cooked rice eating quality value of stored rice − cooked rice eating quality value of fresh rice]/cooked rice eating quality value of fresh rice × 100%.

#### 2.5.2. Comprehensive Evaluation

The storage stability of the eating quality of the rice grains was evaluated based on the method provided by Liu et al. [25] and Zhou et al. [26]. The steps were as follows:

(1) Storage stability score calculation

The storage stability score of the eating quality (appearance or texture or taste) was calculated as follows:

$$r_{i}=\frac{x_{i}-\min x_{i}}{\max{\;x}_{i}-min{\;x}_{i}}\times 10$$

where $r_{i}$ denotes the storage stability score of the eating quality, $x_{i}$ is the variable amplitude of eating quality, and $m\mathrm{ax}x_{i}$ and $\min x_{i}$ represent the maximum and minimum values of each evaluation index, respectively.

(2) Weighted value of the storage stability score of the eating quality

I. Establishing a recursive hierarchical structure

The hierarchical analysis structural model was established based on the interrelationships and affiliations between the indicators of the eating quality of the rice.

II. Constructing a judgment matrix

The judgments were given in numerical form based on the relative importance of each factor at each level (refer to Table 1 for the specific guidelines). Based on this, a judgment matrix (A) was constructed as follows:

$$A={\left(b_{ij}\right)}_{n\times n}$$

III. Calculating the weighted value

The columns of the judgment matrix were normalized:

$${\bar{b}}_{ij}=b_{ij}/\sum_{k=1}^{n}b_{kj}$$

The sum (${\bar{w}}_{i}$) of the data in each row of the normalized judgment matrix was computed:

$${\bar{w}}_{i}=\sum_{j=1}^{n}{\bar{b}}_{ij}$$

The feature vector ($w_{i}$) was obtained by performing a normalization on ${\bar{w}}_{i}$:

$$w_{i}={\bar{w}}_{i}/\sum_{i=1}^{n}{\bar{w}}_{i}$$

The eigenvector ($Aw_{i}$) was calculated using the following formula:

$$Aw_{i}=A\times w_{i}$$

The weight value ($W_{i}$) of the hierarchical element to its subordinate element was calculated by normalizing the eigenvector ($Aw_{i}$).

IV. The consistency test

The largest eigenroot ($\lambda_{max}$) of the judgment matrix was obtained from the following formula:

$$\lambda_{max}=\frac{Aw_{i}}{w_{i}}$$

The consistency index (CI) was calculated according to the following formula:

$$CI=\frac{\lambda_{max}-n}{n-1}$$

The consistency ratio (CR) was derived from the following formula:

CR = CI/RI

where RI is an average stochastic consistency index, and its value is detailed in Table 2. A CR value < 0.10 indicates this judgment matrix had satisfactory consistency. Otherwise, the judgment matrix needed to be adjusted.

(3) Calculating the eating quality storage stability index

The eating quality storage stability index = (storage stability score of cooked rice appearance × weighted value of cooked rice appearance) + (storage stability score of cooked rice texture × weighted value of cooked rice texture) + (storage stability score of cooked rice taste × weighted value of cooked rice taste).

#### 2.5.3. Statistical Analysis

The data were analyzed using Excel 2019 and SPSS 22.0 statistical software. Graphing was conducted using Origin 2021 and the R programming language.

## 3. Results and Discussion

### 3.1. Variable Amplitude

The storage stability of the eating quality of rice varied considerably with the variety. Upon storage, the appearance value of the cooked rice decreased from 6.82 to 5.53, reflecting a variability range of −40.6% to −3.13% and a coefficient of variation of −52.6%. On average, this value dropped by 18.7%. The texture value of the cooked rice declined from 6.47 to 5.24, exhibiting a variability range of −36.1% to −3.56% and a coefficient of variation of −45.8%. This value decreased by an average of 19.1%. The taste value of the cooked rice was reduced from 65.1 to 55.9, showing a variability range of −28.8% to −0.80% and a coefficient of variation of −51.6%. The average decrease in the taste score was 14.2% (Table 3).

During the rice storage, the sulfhydryl groups of proteins at the periphery of the starch in the interior of rice grains undergo oxidation to form disulfide bonds. This process causes the proteins to create a strong reticulation around the starch, which restricts the starch’s ability to swell and reduces its suppleness [27]. Consequently, stored rice tends to be harder and stickier when steamed. Additionally, rice grains stored for longer periods absorb more water during cooking, resulting in longer cooking times compared with those stored for a shorter time [28]. Pasty properties, such as peak viscosity, setback, and final viscosity, show varying changes after storage compared with fresh rice [29]. These variations consequently affect the eating quality. Studies showed that the appearance and taste scores of cooked rice gradually degrade with the rice storage time. This decline in quality is primarily attributed to an increase in the hardness and chewiness of cooked rice, along with a decrease in its elasticity and adhesion. Therefore, following storage, the appearance, texture, and taste of rice when steamed are significantly diminished, leading to a notable reduction in its overall eating quality.

### 3.2. Screening of Rice Varieties

#### 3.2.1. Construction of the Comprehensive Evaluation Index System

As living standards continue to improve alongside economic development, the consumer demand for high-quality rice has significantly increased. The rice that exhibits a glossy appearance and excellent taste after cooking has a clear competitive advantage in the market. However, evaluating the storage stability of the eating quality of rice has become a crucial challenge. The rice eating quality is generally assessed through three indicators: the appearance, texture, and taste of cooked rice [30]. Evaluating the eating quality of different rice varieties cannot be effectively captured by a single index. Therefore, a more comprehensive evaluation system that accounts for multiple aspects of eating quality is essential.

In developing such an evaluation system, previous studies commonly employed methods like principal component analysis, neural networks, and other techniques [25,31]. However, the application effectiveness is inconsistent due to differences in the targets and parameters. For example, principal component analysis can eliminate the correlation effect between evaluation indicators and ensure objectivity. However, its main limitation lies in the fact that the variance contribution rate of the extracted principal components rarely reaches 100%, thus failing to capture all the information of the evaluation object [32].

Hierarchical analysis, proposed by T.L. Saaty, decomposes complex problems into a hierarchical structure and determines the relative importance of indicators through pairwise comparisons. As a multifactorial decision-making approach, it has been applied across various fields [33]. For example, Dr Zhou used hierarchical analysis to assess the rice yield, quality, and nitrogen uptake and utilization under different sowing periods in the lower reaches of the Huai River Basin, identifying temperature and light characteristics that optimize the yield, quality, and efficiency in rice [26]. Dr Zhang utilized hierarchical analysis to evaluate the integrated productivity of wheat in multiple areas in Huaibei, highlighting the critical temperature and light characteristics required for achieving high integrated productivity, which is essential for the production of rice stubble wheat in various wheat areas [34].

**Figure 2:**
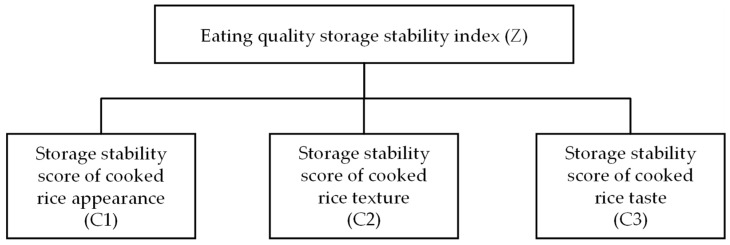
The hierarchical analytical structural model for the comprehensive evaluation of the storage stability of the eating quality of rice.

In this study, hierarchical analysis was adopted to evaluate the storage stability of the eating quality of rice by considering three key dimensions: storage stability score of the cooked rice appearance (C1), storage stability score of the cooked rice texture (C2), and storage stability score of the cooked rice taste (C3). A hierarchical analytical structure for the evaluation of the storage stability was established, as shown in Figure 2. The judgment matrix was constructed based on related theoretical and practical experience, as listed in Table 4. The matrix underwent a consistency test, confirming its reliability (Table 4, CR < 0.10). The weighted values for the storage stability scores of the eating quality of rice were calculated based on Table 4 and the methodology and formulae outlined in Section 2.5.2. The weight values of the appearance, texture, and taste of the cooked rice were 0.105, 0.259, and 0.637, respectively (Table 5).

#### 3.2.2. Eating Quality Storage Stability Index

**Figure 3:**
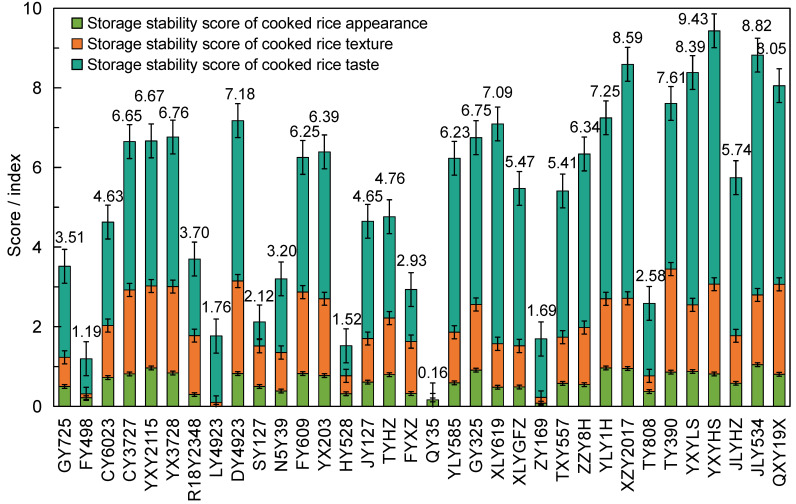
The eating quality storage stability indexes of the tested cultivars.

Normalization is used to eliminate the scale differences by transforming data into a uniform, dimensionless scale [35]. This method is particularly useful for reducing variations in parameters between varieties subjected to different treatments by mapping the data into the (0, 1) interval [36]. In this study, the evaluation indexes for the storage stability of the eating quality of rice were calculated using the normalization method. An evaluation system was established with scores that ranged from 0 to 10. The mean storage stability scores for the three eating quality dimensions were as follows: 5.84 for C1, 5.22 for C2, and 5.21 for C3. The eating quality storage stability index was derived from C1, C2, and C3 and their corresponding weights (Figure 3). The higher the index, the more resistant the rice was to the deterioration in eating quality during storage. For the rice varieties tested in this study, the mean storage stability index of the eating quality was 5.28.

#### 3.2.3. Screening of Rice Varieties with High Storage Stability for Eating Quality

**Figure 4:**
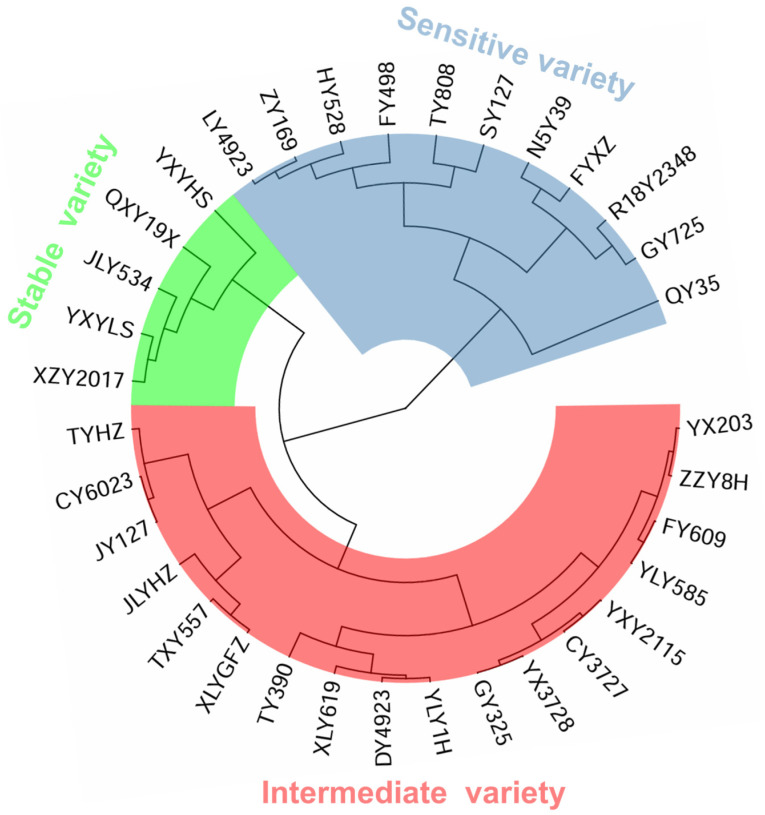
The cluster analysis of storage stability for the eating quality of rice.

A systematic cluster analysis method was employed to categorize the eating quality storage stability indexes of the test varieties. Three types of variety were identified: high storage stability for eating quality (a stable variety), low storage stability for eating quality (a sensitive variety), and intermediate storage stability for eating quality (an intermediate variety). From the cluster analysis, the varieties were grouped as follows: 5 stable rice varieties, 18 intermediate rice varieties, and 11 sensitive rice varieties, accounting for 14.7%, 52.9%, and 32.4% of the total varieties, respectively (Figure 4).

**Figure 5:**
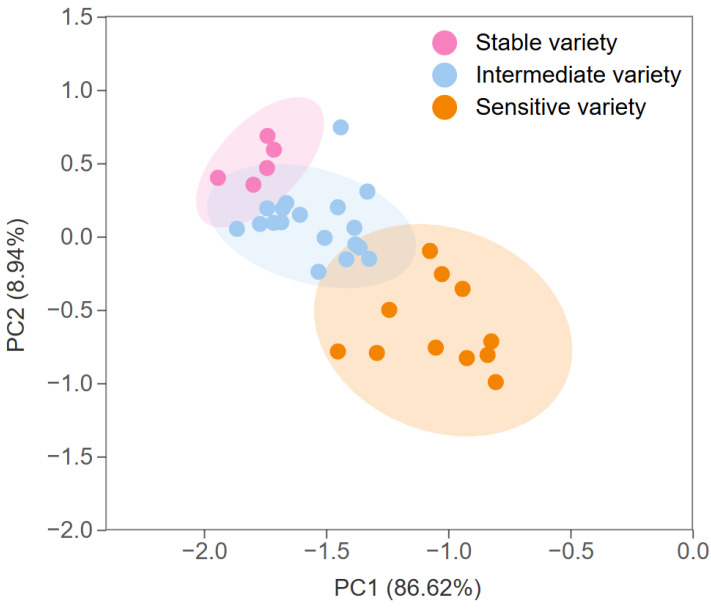
Principal component analysis of different rice varieties.

A principal component analysis was performed to verify the validity and applicability of the three screened categories of varieties with different storage stabilities for the eating quality. The results of the principal component analysis showed that these varieties could be effectively classified into three categories (Figure 5). This result is in general agreement with the results of the cluster analysis, which indicates the reliability of the results of using hierarchical analysis to classify the storage stability for the eating quality of rice.

Although using hierarchical analysis and the judgment matrix for the comprehensive evaluation of the storage stability of the eating quality of rice remains an emerging approach, the results effectively reflect the storage stability of rice varieties. This method represents a promising advancement in improving and refining the screening and evaluation of storage stability for the eating quality of rice. Meanwhile, the identification of five rice varieties with superior eating quality storage stability provides valuable guidance for rice production.

### 3.3. Characteristics of Rice Varieties with High Storage Stability

**Figure 6:**
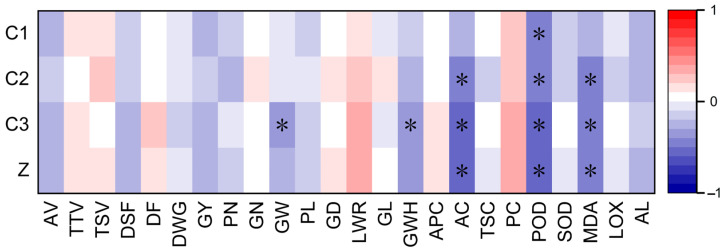
The correlation analysis (*p* < 0.05). Z: eating quality storage stability index, C1: storage stability score of cooked rice appearance, C2: storage stability score of cooked rice texture, C3: storage stability score of cooked rice taste, AV: appearance value of cooked rice, TTV: texture value of cooked rice, TSV: taste value of cooked rice, DSF: days from sowing to flowering, DF: days of filling period, DWG: days of whole growing period, GY: grain yield, PN: panicle number, GN: grain number per panicle, GW: grain weight, PL: panicle length, GD: grain density, LWR: grain length–width ratio, GL: grain length, GWH: grain width, APC: amylopectin starch content, AC: amylose starch content, TSC: total starch content, PC: protein content, POD: peroxidase activity, SOD: superoxide dismutase activity, MDA: malondialdehyde content, LOX: lipoxygenase activity, AL: α-amylase activity. *: significant at *p* < 0.05 levels.

Rice eating quality is generally characterized by the appearance, texture, and taste of cooked rice. However, the analysis found no substantial correlation between the storage stability scores of the cooked rice appearance, texture, and taste; the storage stability index of the eating quality of the rice; and the values of the appearance, texture, and taste of the cooked rice, as shown in Figure 6. This indicates that the ability of rice to withstand eating quality degradation during storage is irrelevant to the intrinsic eating quality of rice.

The period from flowering to maturity is a critical stage for grain filling and enrichment, which has a significant influence on the formation of the initial eating quality of rice [37,38]. However, the eating quality and its storage stability are distinct aspects. Although the flowering-to-maturity process affects the former, it does not necessarily have a direct bearing on the latter. As shown in Figure 6, through correlation analysis, it was found that there was no significant correlation between the storage stability scores of the appearance, texture, and taste of the cooked rice; the eating quality storage stability index; and the days from sowing to flowering, the days of whole growing period, and the days from sowing to flowering. Thus, it can be inferred that the ability of rice to maintain the eating quality during storage was not significantly correlated with the length of the nutritive growth period or the reproductive growth period. It should be emphasized that in this research, all varieties were stored under identical conditions post-harvest to enable a proper investigation of their storage stability characteristics.

As depicted in Figure 6, the eating quality storage stability index was not significantly correlated with rice yield and its components (such as the panicle number, grain number per panicle, and grain weight), panicle shape (including panicle length and grain density), or grain size (such as length–width ratio, grain length, and grain width). Although the correlation analysis indicates that the grain width of rice is related to the storage stability score of cooked rice taste, it had no significant correlation with the storage stability scores of the cooked rice appearance and texture. In particular, it had no significant correlation with the eating quality storage stability index. Consequently, it can be concluded that the grain size of the rice had no significant correlation with the storage stability of the eating quality of the rice.

Starch and protein are the primary components of rice grains. Starch consists of amylose and amylopectin [39]. The storage stability of the rice was significantly and negatively correlated with the amylose starch content but not significantly related to the amylopectin starch content, total starch content, and protein content (Figure 6). During storage, the structure of the amylose starch in rice grains might change, thus affecting pasting properties [5]. Rice varieties with lower amylose starch content tend to experience less structural alteration in amylose starch after storage, resulting in more stable pasting properties and improved eating quality [40]. Table 6 shows that the amylose starch content of the stable variety was 12.4% lower than that of the sensitive variety. This demonstrates that the characteristics of rice varieties with high storage stability for eating quality include a low amylose starch content.

Superoxide dismutase activity, peroxidase activity, malondialdehyde content, lipoxygenase activity, and α-amylase activity were identified as factors that profoundly influence the storage stability of rice [41]. In this study, the storage stability was found to be significantly and negatively correlated with the peroxidase activity and malondialdehyde content but not significantly linked to the superoxide dismutase activity, lipoxygenase activity, and α-amylase activity (Figure 6).

The peroxidase activity is a critical indicator of the freshness and storage stability of rice grains. It catalyzes the decomposition of hydrogen peroxide and other organic peroxides in rice grains [42]. A lower level of peroxidase activity indicates a slower rate of oxidative reactions in rice grains during storage, leading to a corresponding reduction in oxidation reactions. This reduction helps prevent the formation of undesirable compounds that negatively affect the appearance, texture, and taste of cooked rice, thereby maintaining a more stable eating quality in the rice. As shown in Table 6, the stable variety exhibited a 35.9% lower peroxidase activity than the sensitive variety. It can be concluded that one of the characteristics of rice varieties with high storage stability for eating quality is lower peroxidase activity.

Malondialdehyde is a product of lipid peroxidation and is commonly used as a biomarker for oxidative stress and cellular damage [43]. During storage, lipids in rice grains undergo peroxidation due to environmental factors, such as oxygen, temperature, and humidity [44]. This process is a free radical chain reaction: unsaturated fatty acids are attacked by active oxygen species (superoxide anions and hydroxyl radicals), forming lipid radicals. Then, these lipid radicals react with oxygen to generate lipid peroxides, which decompose to form malondialdehyde [11,45]. A low malondialdehyde content in rice grains implies a relatively low lipid peroxidation, suggesting that the fats in rice grains are well-preserved and not excessively subjected to oxidative damage, and lipids are not susceptible to oxidative reactions. Lipid oxidation produces undesirable compounds that deteriorate the eating quality of rice [46]. Therefore, maintaining a low malondialdehyde content in rice during storage contributes to the preservation of its eating quality after cooking. Table 6 shows that the malondialdehyde content of the stable rice variety was 6.42% lower than that of the sensitive variety. Therefore, one of the characteristics of rice varieties with high storage stability for eating quality is a low malondialdehyde content.

Different storage conditions play a significant role in determining the rate of deterioration of rice eating quality. Storage conditions can either hasten or decelerate the metabolic and physiological processes during rice storage [14]. For example, studies showed that a high temperature and high humidity can accelerate the metabolism and quality changes of rice seeds [47]. Under such conditions, several key changes occur. First, the free fatty acid content increases remarkably, and peroxides are formed due to lipid oxidation [17]. Second, the catalase activity decreases, leading to the accumulation of peroxides [15]. Third, the levels of volatile compounds, like aldehydes, ketones, and furans, rise, all of which contribute to the degradation of the rice eating quality [48]. In addition, the gas composition of the storage environment also has a major impact on rice grain storage. Controlled atmosphere storage can inhibit rice respiration and extend the storage life [49]. In the case of rice stored in carbon dioxide-controlled atmosphere storage, the degree of fatty acid change, cooked rice hardness, lipoxygenase activity, and α-amylase activity are all lower than those in conventional storage (in air) [50]. Nitrogen and carbon dioxide-controlled atmosphere storage slow down the changes in macromolecular properties and maintain the proportion of surface starch/lipid/protein during a six-month storage period [51].

In this study, the 34 selected samples were all subjected to the same treatment process. After the harvest, they were separated from impurities and then naturally dried. Subsequently, each sample was placed in an individual mesh bag and hung for storage in a warehouse. During the storage period, the temperature and humidity of all samples were consistent. This uniformity of storage conditions ensured the reliability of our research. This allowed us to focus on the inherent characteristics of the rice varieties and their relationship with the eating quality during storage, without the interference of variable storage conditions. This, in turn, provided a solid foundation for analyzing the stability of the eating quality of different rice varieties and exploring the key factors associated with it.

## 4. Conclusions

Screening for rice varieties with stable eating quality during storage is crucial for ensuring grain safety and quality preservation. This study conducted a comprehensive investigation into the eating quality stability for 34 widely cultivated rice varieties during one year of natural storage. The research indicated that the appearance, texture, and taste of the cooked rice declined by 18.7%, 19.1%, and 14.2%, respectively, after storage. Using a hierarchical analysis, normalization method, and cluster analysis, five rice varieties with high eating quality storage stability were screened out. Importantly, the key traits associated with high eating quality storage stability in rice varieties were found to be a low amylose starch content, reduced peroxidase activity, and low malondialdehyde content. Overall, this research not only enriches our comprehension of the intricate relationship between the storage stability of the eating quality of rice and its physiological indicators, but also paves the way for formulating effective strategies for the rice safe storage and the targeted breeding of rice varieties with enhanced eating quality storage stability. Consequently, it makes a substantial and far-reaching contribution to the sustainable development and optimization of the rice industry.

## Figures and Tables

**Table 1:** The scales and their meanings.

Scale	Meaning
1	Equal importance of both indicators
3	Slightly more important for one indicator compared with the other
5	Noticeably more important for one indicator compared with the other
7	Extremely more important for one indicator compared with the other
2, 4, 6	The median of two adjacent judgments

**Table 2:** The RI value.

Order n	1	2	3	4	5	6	7	8	9
RI value	0.00	0.00	0.58	0.90	1.12	1.24	1.32	1.41	1.45

**Table 3:** The variable amplitude of the eating quality of rice after storage.

	Range	Mean Value	CV (%)
The appearance value of fresh rice after cooking	(5.70, 7.75)	6.82	6.98
The appearance value of stored rice after cooking	(3.70, 6.55)	5.50	12.3
Variable amplitude of the appearance value (%)	(−40.6, −3.13)	−18.7	−52.6
The texture value of fresh rice after cooking	(5.20, 7.35)	6.47	7.88
The texture value of stored rice after cooking	(3.55, 6.20)	5.24	13.5
Variable amplitude of the texture value (%)	(−36.1, −3.56)	−19.1	−45.8
The taste value of fresh rice after cooking	(55.5, 74.3)	65.1	6.66
The taste value of stored rice after cooking	(43.1, 67.2)	55.9	10.9
Variable amplitude of the taste value (%)	(−28.8, −0.80)	−14.2	−51.6

Note: CV—coefficient of variation.

**Table 4:** The judgment matrix and consistency test.

Z	C1	C2	C3
C1	1	1/3	1/5
C2	3	1	1/3
C3	5	3	1

Note: $\lambda_{max}$ = 3.04, CI = 0.019, RI = 0.58, CR = 0.033 < 0.10; Z: eating quality storage stability index, C1: storage stability score of cooked rice appearance, C2: storage stability score of cooked rice texture, C3: storage stability score of cooked rice taste.

**Table 5:** Weight values of the comprehensive evaluation indicators for the storage stability of the eating quality of rice.

	Storage Stability of Cooked Rice Appearance	Storage Stability of Cooked Rice Texture	Storage Stability of Cooked Rice Taste
Weight value	0.105	0.259	0.637

**Table 6:** The key characteristics of rice varieties with a high storage stability.

Variety Type	Index	Mean Value	Variable Amplitude (%)	CV (%)
Stable variety	Amylose starch content (%)	17.0	(16.3, 17.7)	3.07
	Peroxidase activity (U/g)	18.4	(12.6, 25.8)	23.9
	Malondialdehyde content (%)	3.23	(2.90, 3.38)	5.70
Intermediate variety	Amylose starch content (%)	17.8	(15.4, 19.8)	7.22
	Peroxidase activity (U/g)	23.1	(13.8, 31.1)	19.0
	Malondialdehyde content (%)	3.30	(2.49, 3.55)	6.43
Sensitive variety	Amylose starch content (%)	19.4	(15.3, 22.9)	13.2
	Peroxidase activity (U/g)	28.7	(14.0, 41.8)	23.5
	Malondialdehyde content (%)	3.45	(3.23, 3.66)	4.55

Note: CV—coefficient of variation.

## Data Availability

The data presented in this study are available on request from the corresponding author. The data are not publicly available due to privacy restrictions.

## Supplementary Materials

The following supporting information can be downloaded from https://www.mdpi.com/article/10.3390/foods13244140/s1—Table S1: Experimental materials.
