# Supplementary material for: Variety Screening and Characterization Analysis of Storage Stability of Eating Quality of Rice
Source: Foods. 2024 Dec 20;13(24):4140. doi: 10.3390/foods13244140 (PMC11675847; doi:10.3390/foods13244140)
Supplement: Supplementary file 1 [file foods-13-04140-s001.zip › foods-3355724-supplementary.pdf]

Supplemental Table S1 Experimental materials

Chinese variety name	English variety name	Abbreviation
冈优 725	Gangyou 725	GY725
F 优 498	F you 498	FY498
川优 6203	Chuanyou 6203	CY6023
川优 3727	Chuanyou 3727	CY3727
宜香优 2115	Yixiangyou 2115	YXY2115
宜香 3728	Yixiang 3728	YX3728
蓉 18 优 2348	Rong 18 you 2348	R18Y2348
绿优 4923	Lvyou 4923	LY4923
德优 4923	Deyou 4923	DY4923
蜀优 127	Shuyou 127	SY127
内 5 优 39	Nei 5 you 39	N5Y39
繁优 609	Fanyou 609	FY609
渝香 203	Yuxiang 203	YX203
花优 528	Huayou 528	HY528
旌优 127	Jingyou 127	JY127
天优华占	Tianyouhuazhan	TYHZ
丰优香占	Fengyouxiangzhan	FYXZ
黔优 35	Qianyou 35	QY35
Y 两优 585	Y liangyou 585	YLY585
G 优 325	G you 325	GY325
香两优 619	Xiangliangyou 619	XLY619
香两优贵福占	Xiangliangyouguifuzhan	XLYGFZ
中优 169	Zhongyou 169	ZY169
T 香优 557	T xiangyou 557	TXY557
中浙优 8 号	Zhongzheyou No.8	ZZY8H
Y 两优 1 号	Y liangyou No.1	YLY1H
香早优 2017	Xiangzaoyou 2017	XZY2017
泰优 808	Taiyou 808	TY808
泰优 390	Taiyou 390	TY390
野香优莉丝	Yexiangyoulisi	YXYLS
野香优海丝	Yexiangyouhaisi	YXYHS
晶两优华占	Jingliangyouhuazhan	JLYHZ
晶两优 534	Jingliangyou 534	JLY534
青香优 19 香	Qingxiangyou 19 xiang	QXY19X
